# Comparison of Pandemic (H1N1) 2009 and Seasonal Influenza Viral Loads, Singapore

**DOI:** 10.3201/eid1702.100282

**Published:** 2011-02

**Authors:** Chun K. Lee, Hong K. Lee, Tze P. Loh, Florence Y.L. Lai, Paul A. Tambyah, Lily Chiu, Evelyn S.C. Koay, Julian W. Tang

**Affiliations:** Author affiliations: National University Hospital, Singapore (C.K. Lee, H.K. Lee, T.P. Loh, P.A. Tambyah, L. Chiu, E.S.C. Koay, J.W. Tang);; Ministry of Health, Singapore (F.Y.L. Lai);; National University of Singapore, Singapore (P.A. Tambyah, E.S.C. Koay)

**Keywords:** Influenza, pandemic, seasonal, H1N1, viral load, clinical severity, comorbidities, viruses, Singapore, dispatch

## Abstract

Mean viral loads for patients with pandemic (H1N1) 2009 were ≈1 log_10_ times lower than those for patients with seasonal influenza within the first week after symptom onset. Neither pandemic nor seasonal influenza viral loads correlated with clinical severity of illness. No correlation was found between viral loads and concurrent illness.

Although clinical characteristics of pandemic (H1N1) 2009 have been well documented (*1**,**2*), fewer specific virologic comparisons with seasonal influenza have been studied in hospitalized patients (*3*). Studies of other influenza virus infections in humans suggest that host immune responses play a major role in determining clinical outcomes (*4**,**5*). We describe the initial viral loads for patients infected with pandemic (H1N1) 2009 and seasonal (H1 and H3) influenza viruses and their correlation with various aspects of signs and symptoms at admission to the National University Hospital (NUH) in Singapore.

## The Study

The study consisted of patients seen at NUH during May–November 2009 as emergency admissions, outpatients, or inpatients whose nasopharyngeal swabs submitted for routine diagnostic testing were positive for seasonal influenza virus A (H1 and H3) or pandemic influenza A virus (H1N1) 2009. From samples taken before treatment was begun, we identified 578 patients with pandemic (H1N1) 2009 and 88 patients with seasonal influenza (11 H1 and 77 H3). Clinical characteristics of some of these patients have been described elsewhere (*2*). Local ethics approval (ref. no. B/09/360) was granted for this study.

Age, sex, and clinical information (i.e., days after onset of symptoms, comorbidities, clinical severity) were obtained from patient records. Comorbidities were defined as >1 of the conditions listed in Table 1. Clinical severity was defined as follows: mild, patients well enough to be treated as outpatients; moderate, patients ill enough to warrant hospital admission; severe, hospitalized patients who died or who required intensive or high-dependency care. In-house quantitative assays (Technical Appendix) were performed on archived samples previously tested as positive for pandemic (H1N1) 2009 and reported elsewhere (*6*).

**Table 1:** Comparison of baseline characteristics between patients with pandemic (H1N1) 2009 and seasonal influenza H3 infection, Singapore, May–November 2009

Characteristic	Pandemic (H1N1) 2009, no. (%), n = 578	Seasonal influenza H3, no. (%), n = 77	p value
Age, y			<0.0001
0–4	69 (11.9)	7 (9.1)	
5–14	144 (24.9)	11 (14.3)	
15–34	250 (43.3)	28 (36.4)	
35–54	72 (12.5)	13 (16.9)	
>55	43 (7.4)	18 (23.4)	
Female sex	275 (47.6)	41 (53.2)	0.3959
Comorbidities*	262 (45.3)	22 (28.6)	0.0068
Asthma	120 (20.8)	7 (9.1)	0.0137
Chronic lung disease	15 (2.6)	3 (3.9)	0.4584
Cardiac disease	21 (3.6)	4 (5.2)	0.5214
Chronic renal failure	21 (3.6)	2 (2.6)	1.0000
Chronic liver disease	11 (1.9)	0	0.6275
Cerebrovascular disease	9 (1.6)	2 (2.6)	0.3776
Neoplasms	22 (3.8)	3 (3.9)	1.0000
Diabetes	41 (7.1)	5 (6.5)	1.0000
Pregnancy	39 (6.7)	2 (2.6)	0.2115
Immunocompromised	27 (4.7)	2 (2.6)	0.5621
Receipt of steroid medication	23 (4.0)	1 (1.3)	0.3429
Autoimmune disease	14 (2.4)	1 (1.3)	1.0000
Neurocognitive disease	12 (2.1)	1 (1.3)	1.0000
Neuromuscular disease	2 (0.3)	0	1.0000
Premitigation phase	104 (18.0)	51 (66.2)	<0.0001
Clinical severity†			0.0462
Severe cases‡	23 (4.9)	1 (3.8)	
Hospitalized cases§	222 (46.8)	6 (23.1)	
Outpatient only	229 (48.3)	19 (73.1)	

*Patient had >1 of the conditions listed. †Analysis was limited to patients in whom influenza were diagnosed during the mitigation phase (n = 474 for pandemic and n = 26 for H3 seasonal influenzas). Singapore switched from premitigation (i.e., containment) to mitigation management protocols on July 8, 2009, which altered how patient treatment with oseltamivir was initiated. However, this transition does not affect the results shown above because none of the patients were undergoing treatment when these first diagnostic samples were taken. ‡Patients requiring intensive or high-dependency care or who died. §Patients requiring hospitalization because of clinical conditions but not intensive or high-dependency care.

**Figure 1:**
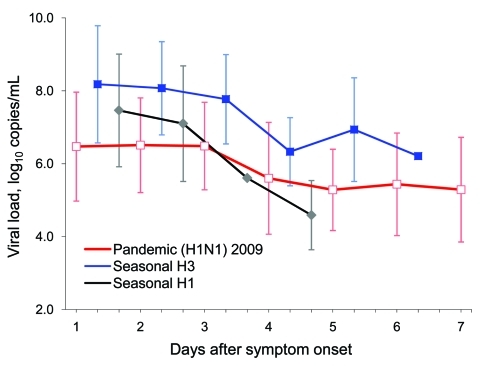
Viral loads (in RNA copies/mL) in patients with pandemic (H1N1) 2009 (NP) and seasonal H1 and H3 (MP) influenza at time patient sought hospital care against days after symptom onset. Vertical bars indicate ±1 SD. Line plots are slightly offset with respect to each other along the time axis to allow the SD bars to be seen clearly. NP, nucleoprotein; MP, matrix protein.

Viral loads of hemagglutinin (HA) and nucleoprotein (NP) for pandemic (H1N1) 2009 ranged from 10^2^ to 10^9^ RNA copies/mL of virus transport medium (mean 10^5^–10^7^ RNA copies/mL). Seasonal influenza viral loads ranged from 10^3^ to 10^10^ RNA copies/mL (mean 10^6^–10^8^ RNA copies/mL for seasonal influenza subtype H3 and mean 10^5^ to 10^7^ RNA copies/mL for seasonal influenza H1). Viral loads decreased with time after onset of symptoms from date the patient sought care at NUH in patients with pandemic or seasonal influenza (Figure 1).

Because of the small number of patients with seasonal influenza H1, further analysis for seasonal influenza was limited to H3. Patients infected with pandemic (H1N1) 2009, compared with those having seasonal influenza H3, were younger (p<0.0001), and a higher proportion had comorbidities (p = 0.0068; Table 1).

**Figure 2:**
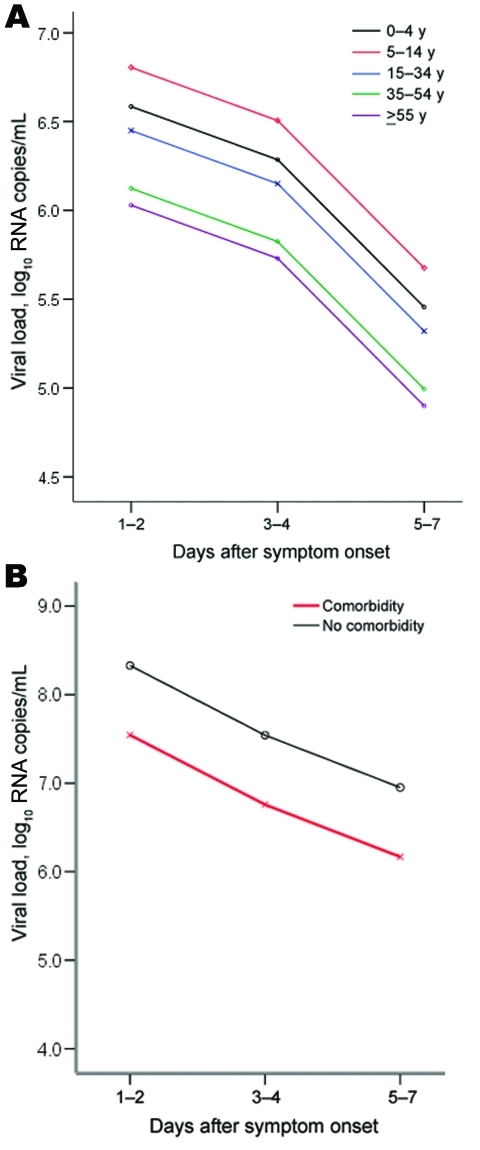
A) Profile plot and multivariate comparisons of the estimated nucleoprotein viral loads of pandemic (H1N1) 2009, by patient age group, against days from symptom onset in the final multiple analysis of variance model. B) Profile plot and comparisons of the estimated matrix protein viral loads of seasonal influenza H3 by the presence or absence of comorbidities against days from symptom onset in the final analysis of variance model.

For the 578 pandemic influenza cases, the multiple analysis of variance showed that viral loads were associated with number of days after symptom onset from date of presentation (p<0.0001) and with age (p = 0.0112) (Figure 2, panel A; Table 2). For the 77 seasonal influenza H3 cases, the analysis of variance showed that days after onset of symptoms from date of presentation (p = 0.0223) and presence of any comorbidities (p = 0.0249) significantly affected viral loads (Table 2). Viral loads for seasonal influenza were lower in patients with than without comorbidities (Figure 2, panel B).

**Table 2:** Analysis of pandemic (H1N1) 2009 (HA and NP) and seasonal H3 (MP) viral loads with clinical parameters, Singapore, May–November 2009*

Characteristic	Pandemic influenza					Seasonal H3 influenza		
	No.	HA viral load, log_10_ copies/mL, mean (SD)	NP viral load, log_10_ copies/mL, mean (SD)	MANOVA p value†		No.	MP viral load, log_10_ copies/mL, mean (SD)	ANOVA p value
Time from symptom onset, d				<0.0001				0.0223
1–2	416	6.49 (1.44)	6.49 (1.38)			53	8.12 (1.43)	
3–4	114	6.18 (1.39)	6.16 (1.40)			20	7.27 (1.31)	
5–7	48	5.33 (1.21)	5.31 (1.23)			4	6.76 (1.22)	
Age, y				0.0112‡				0.9652‡
0–4	69	6.46 (1.40)	6.45 (1.39)			7	7.66 (0.75)	
5–14	144	6.62 (1.36)	6.65 (1.26)			11	7.93 (1.38)	
15–34	250	6.34 (1.48)	6.33 (1.43)			28	7.88 (1.66)	
35–54	72	5.85 (1.46)	5.88 (1.41)			13	7.99 (1.04)	
>55	43	5.88 (1.41)	5.83 (1.50)			18	7.63 (1.66)	
Sex				0.3018‡				0.3883‡
F	275	6.23 (1.49)	6.26 (1.38)			41	7.68 (1.52)	
M	303	6.42 (1.41)	6.39 (1.43)			36	8.00 (1.35)	
Comorbidities				0.9967‡				0.0249‡
Yes	262	6.35 (1.49)	6.35 (1.44)			22	7.23 (1.53)	
No	316	6.31 (1.42)	6.31 (1.39)			55	8.07 (1.35)	
Clinical severity§								
Severe	23	5.97 (1.76)	5.98 (1.84)					
Hospitalized	222	6.44 (1.49)	6.42 (1.43)			7¶	7.55 (1.06)	
Outpatient	229	6.29 (1.45)	6.30 (1.40)			19	7.25 (1.54)	

*HA, hemagglutinin; NP, nucleoprotein; MP, matrix protein; MANOVA, multiple analysis of variance; ANOVA, analysis of variance. †Wilks Lambda statistics. ‡Effect of days from symptom onset adjusted. §Analysis included patients who sought care during the mitigation phase only (n = 474 for pandemic and n = 26 for H3 seasonal influenzas). Singapore switched from premitigation (i.e., containment) to mitigation management protocols on July 8, 2009, which altered how patient treatment with oseltamivir was initiated. However, this transition does not affect the results shown above because none of the patients were undergoing treatment when these first diagnostic samples were taken. ¶Includes 1 severe case.

## Conclusions

One of our most striking findings was that the mean viral loads of patients visiting NUH were ≈1 log_10_ higher for seasonal than for pandemic influenza (Figure 1). This difference persisted even after we adjusted for age. Another study demonstrated that within the first 3 days after symptom onset, historical mean viral loads of seasonal influenza exceed those of the contemporary pandemic virus by 1–2 log_10_ (*3*). However, a limitation of that study is its use of viral load data for seasonal influenza that was historical rather than obtained contemporaneously with the data for pandemic (H1N1) 2009.

Approximately 30%–50% of influenza case-patients may be asymptomatic (*7*), and although the correlation between viral load and clinical symptoms is not well established, a viral load threshold may exist below which most persons have no clinical symptoms (although individual variation will always exist). Our analysis suggests that if such a threshold exists, it is lower for novel than for seasonal influenza viruses. For a direct virus-mediated pathologic process, this hypothesis may be understandable, given the lower prevalence of preexisting (and therefore potentially partially protective) cross-reactive immunity for this novel virus (*8**–**10*).

Viral loads for both pandemic (H1N1) 2009 and seasonal influenza tend to decrease with time after symptom onset (Figure 1). Larger studies are needed to confirm the more rapid decline of seasonal influenza H1 than of H3 viral loads. In addition, younger age groups had significantly higher viral loads for pandemic (H1N1) 2009 (Figure 2, panel A), which may not be surprising given that this Southeast Asian population appears to have little or no preexisting specific or cross-reacting antibodies to this novel virus (*9**,**10*).

Two findings are perhaps the most surprising of this analysis. First, we found no significant correlation between pandemic (H1N1) 2009 or seasonal influenza viral loads and clinical severity of illness (Table 2). Second, pandemic (H1N1) 2009 viral loads in infected patients with and without preexisting comorbidities did not differ significantly, although a significant difference was found for seasonal influenza (Figure 2, panel B; Table 2). We offer some possible explanation for these findings but note that these influenza viral loads have been measured in respiratory samples. These samples are peripheral types of specimens that may not necessarily directly affect, or be directly affected by, many of the preexisting comorbidities that involve nonrespiratory systems, unless their management involves, for example, some sort of immunosuppressive therapy.

A main limitation of this study is that these viral load measurements were performed on only 1 acute diagnostic sample from each patient at admission before treatment with oseltamivir; therefore, determining how these viral loads would have changed later during the natural course of the infection was not possible. Also, some of the patient categories (Table 1, Table 2) contained relatively few patients, e.g., the relatively low number of severe cases (Table 1, Table 2), which may have limited the statistical significance of some correlations. Finally, although influenza viral loads in various types of respiratory samples are now often reported (*3**,**6*), these are heterogeneous, peripheral samples, and such viral loads may vary considerably in the same patient during a single day, depending on individual host immune responses.

If human illness caused by influenza virus infections is mediated by host immune responses (*4**,**5*), then a more vigorous, primary immune response in the immunologically naive, otherwise healthy younger population against the pandemic (H1N1) 2009 virus may also contribute to the degree of clinical illness. The interplay between a direct viral pathologic process and a host immune-mediated pathologic process is probably unique to each person. Some recent studies investigating cytokine responses in persons with acute pandemic (H1N1) 2009 infections had contrasting findings (*11**–**13*), although postmortem investigations of some fatal cases of pandemic (H1N1) 2009 infection found substantial inflammation, which supports an immune-mediated pathologic process for at least in these cases (*14*). Similarly, for the more well-established seasonal H3 influenza (to which most persons have had many years of exposure) more well-established, robust, yet sufficiently individually different patterns of homologous and heterologous immune responses may contribute more (compared with similar responses to pandemic [H1N1] 2009) to the different degrees of clinical illness in infected persons with different combinations of comorbidities.

## Supplementary Material

Appendix Additional Laboratory Methods and Statistical Analysis.
