# Supplementary material for: Comparison of Pandemic (H1N1) 2009 and Seasonal Influenza Viral Loads, Singapore
Source: Emerg Infect Dis. 2011 Feb;17(2):287–90. doi: 10.3201/eid1702.100282 (PMC3204747; doi:10.3201/eid1702.100282)
Supplement: Appendix — Additional Laboratory Methods and Statistical Analysis. [file 10-0282-Techapp.pdf]

Article DOI: 10.3201/eid1702.100282

Comparison of Pandemic (H1N1) 2009 and Seasonal Influenza Viral Loads, Singapore

Technical Appendix

Additional Laboratory Methods

All the archived nasopharyngeal swabs samples used in this study were originally collected for diagnostic testing (archived as the original virus transport medium specimen containing live virus). These samples have been stored at –80°C with no further freeze-thawing for additional testing. These were thawed for the first time and tested in these quantitative assays as a series of large batches over several days.

The initial influenza screening protocol applied to each sample and the details of the specific qualitative RT-PCR assays for detecting influenza A and the subsequent subtyping to seasonal H1 or H3 or pandemic (H1N1) 2009 have been described elsewhere *(1)*. In addition, the quantitation of the pandemic (H1N1) 2009 is also detailed in this publication *(1)*.

Brief methodologic details of the quantitation of the seasonal influenza virus subtypes (H1 and H3) described in this study are given below as the details of this assay are currently being submitted for publication elsewhere (further details are available from the authors, upon request, until this manuscript has been accepted).

Quantitation of the seasonal (H1 and H3) viruses for this study was performed by using an in-house assay that targeted the matrix protein (MP) gene sequence. The conserved MP region that was targeted, theoretically (in silico), allowed the detection of all human influenza subtypes. Prequantified, in-house calibrators (i.e., cloned plasmids covering the PCR gene target regions and/or in vitro transcribed RNAs) were included in each assay run, to allow the construction of run-specific calibration curves to accurately quantify the viral loads in each of the archived samples for this study.

Technical Appendix Table 1. Primer sequences and cycling condition of the universal influenza A assay

Name	Primer and probe sequences	Gene target	Orientation	5’ Position*
Universal influenza A	5’-GGA ATG GCT AAA GAC AAG ACC AAT-3’	Matrix	Forward	119
	5'-GGG CAT TTT GGA CAA AGC GTC TAC-3'		Reverse	240
	5'-(FAM) AGT CCT CGC TCA CTG GGC ACG GTG (BHQ1)-3'		Reverse	211

*Location of the oligonucleotides based on the strain of pandemic influenza A/2009/H1N1, Genbank accession no. FJ966085.

PCR Thermal cycling conditions

Step	Temperature	Time
Reverse transcription	55°C	8 min
Initial denaturation	95°C	2.5 min
Amplification (45 cycles)	95°C	15 s
	60°C	20 s
	68°C*	15 s

*Fluorescent signals taken for data acquisition.

Statistical Analysis

Viral load concentrations (copies/mL) were log-transformed (base 10) for statistical analyses. Owing to the very small numbers of H1 cases diagnosed during this period, further analysis for seasonal influenza limits to seasonal H3 only. Comparisons of the baseline characteristics (demographics and underlying diseases) between patients with pandemic (H1N1) 2009 and seasonal influenza H3 were performed with the Chi-square test or Fisher exact test, where appropriate. Among patients with pandemic (H1N1) 2009, association between the HA and NP viral loads and patient characteristics (demographics, underlying diseases, and clinical severity of illness) were assessed with the multivariate analysis of variance. For seasonal influenza H3, associations between the viral loads (MP) and patient characteristics were assessed with analysis of variance. All statistical tests were performed by using SAS software version 9.0 (SAS, Carey, NC, USA); a p value of <0.05 was considered statistically significant.

Reference

1. Lee HK, Lee CK, Loh TP, Tang JW, Chiu L, Tambyah PA, et al. Diagnostic testing for pandemic influenza in Singapore: a novel dual-gene quantitative real-time RT-PCR for the detection of influenza A/H1N1/2009. J Mol Diagn. 2010;12:636–43. PubMed DOI:10.2353/jmoldx.2010.100010
